# Vaccination with MVA/HIV induces differential recruitment of monocyte subsets into the circulation and monocyte-specific transcriptional programs

**DOI:** 10.1186/1742-4690-9-S2-O18

**Published:** 2012-09-13

**Authors:** E Andersen-Nissen, DE Zak, TR Hensley, DJ Adams, X Hu, A Sato, M Elizaga, PA Goepfert, HL Robinson, A Aderem, MJ McElrath

**Affiliations:** 1 Fred Hutchinson Cancer Research Center, Seattle, WA, USA; 2 Seattle BioMed, Seattle, WA, USA; 3 University of Alabama, Birmingham, AL, USA; 4 GeoVax, Smyrna, GA, USA

## Background

Monocyte subpopulations are recruited from marrow to blood in response to infection and migrate into inflamed tissues to initiate adaptive immune responses. We sought to understand the effect of an MVA-vectored HIV vaccine on peripheral blood monocytes within the first week post-vaccination.

## Methods

Volunteers were vaccinated with MVA/HIV62 gag/pol/env (protocol HVTN 205/908); blood was collected pre-vaccination and 1, 3, and 7 days post-vaccination. Serum cytokine analysis was performed (n=13). Monocytes were phenotyped by flow cytometry (n=14) and microarray transcriptional profiling was performed on freshly-sorted monocytes (n=7).

## Results

MVA/HIV induced serum elevations of the monocyte chemoattractant CCL2, and seven proinflammatory cytokines at 24 hours post-vaccination (p<0.001). Two waves of monocyte population changes occurred: concomitant with the rise in CCL2, CD14^++^ CD16_-_ ‘classical’ monocyte concentrations increased at 24 hours post-vaccination (p=0.002). In contrast, CD14^++^CD16^+^ ‘intermediate’ and CD14^+^CD16^++^ ‘patrolling’ monocyte concentrations did not increase until 72 hours post-vaccination (p< 0.001). Vaccination induced expression of the activation markers CD86 and HLA-DR at the 72 hour timepoint on all subpopulations examined (p<0.01), indicating a phenotypic shift towards increased antigen presentation. Microarray analysis of sorted CD14^+^ monocytes (encompassing all three subpopulations) revealed that the majority of gene expression changes occurred at 24 hours (494 up- and 53 down-regulated genes; FDR≤10% and average absolute log _2_ (fold-change) ≥0.5, including antigen-processing genes (PSMA2, 3, 5), IL-6, and TLR-pathway genes (TLR1, LY96). Only 39 genes were up-regulated at 72 hours post-vaccination. 351/547 genes regulated at 24 hours were not observed in bulk PBMC profiles from the same individuals, indicating the value of early profiling of monocyte subpopulations.

## Conclusion

These data provide the most detailed profile of the human in vivo monocyte response to an MVA poxviral vector to date. Analyses of transcriptional signatures that predict T-cell and antibody responses will enable the identification of innate immune response genes to target with future HIV vaccines.

